# A complex network of transcription factors and epigenetic regulators involved in bovine leukemia virus transcriptional regulation

**DOI:** 10.1186/s12977-023-00623-w

**Published:** 2023-06-02

**Authors:** Estelle Plant, Maxime Bellefroid, Carine Van Lint

**Affiliations:** Service of Molecular Virology, Department of Molecular Biology (DBM), Université Libre de Bruxelles (ULB), 6041 Gosselies, Belgium

**Keywords:** Bovine Leukemia Virus, Latency, Transcription, Epigenetics

## Abstract

Bovine Leukemia Virus (BLV) is the etiological agent of enzootic bovine leukosis, a disease characterized by the neoplastic proliferation of B cells in cattle. While most European countries have introduced efficient eradication programs, BLV is still present worldwide and no treatment is available. A major feature of BLV infection is the viral latency, which enables the escape from the host immune system, the maintenance of a persistent infection and ultimately the tumoral development. BLV latency is a multifactorial phenomenon resulting in the silencing of viral genes due to genetic and epigenetic repressions of the viral promoter located in the 5ʹ Long Terminal Repeat (5ʹLTR). However, viral miRNAs and antisense transcripts are expressed from two different proviral regions, respectively the miRNA cluster and the 3ʹLTR. These latter transcripts are expressed despite the viral latency affecting the 5ʹLTR and are increasingly considered to take part in tumoral development. In the present review, we provide a summary of the experimental evidence that has enabled to characterize the molecular mechanisms regulating each of the three BLV transcriptional units, either through *cis*-regulatory elements or through epigenetic modifications. Additionally, we describe the recently identified BLV miRNAs and antisense transcripts and their implications in BLV-induced tumorigenesis. Finally, we discuss the relevance of BLV as an experimental model for the closely related human T-lymphotropic virus HTLV-1.

## Background

Bovine Leukemia Virus (BLV) is a B-lymphotropic retrovirus naturally infecting cattle. In the majority of the cases, infected animals remain lifelong asymptomatic carriers, while ~ 30% will present a persistent lymphocytosis, characterized by an accumulation of infected B lymphocytes [1]. In a minority of cases (less than 5%), infected animals can develop B-cell lymphoma or leukemia termed enzootic bovine leukosis (EBL) after a long incubation period of on average 7 years [2, 3]. Experimentally, several other species are permissive to infection such as rabbits, rats, chickens, pigs, goats and sheep, but only goats and sheep develop a persistent infection which is able to induce tumoral development [4]. Interestingly, sheep are highly sensitive to BLV infection and the majority (> 95%) will develop EBL after a shorter period of incubation ranging from 1 to 4 years [1], thus constituting a convenient model to study the mechanisms of BLV-induced tumoral development [4–10]. Although now eradicated in most European countries through the introduction of costly sanitary rules, consisting mainly in the testing and slaughtering of BLV-infected animals, BLV is still highly present in the USA and other regions where it is associated with important economic losses [11, 12]. Condemnation of carcasses showing evidence of tumoral development, which are not authorized to enter the human food chain, is the main source of these losses. In the USA, BLV-induced tumoral development constitutes up to 20% of total condemnations in slaughter plants [13, 14]. Additionally, other side effects linked to BLV infection, such as reduced lifespan of infected dairy cattle, reduction in milk production, increased susceptibility to opportunistic pathogens, and trade restrictions imposed on infected cattle and their products, are also causes of important economic losses [11, 15]. Currently, the most efficient method for controlling BLV infection is the systematic test of the herds and the culling or segregation of BLV-positive animals. However, where BLV prevalence is high, this method is not economically feasible and preventive methods avoiding the transfer of infected body fluids are mostly used [11]. To date, although several strategies such as treatment with the histone deacetylase inhibitor valproate [16] have been tested, none of them has proved sufficient efficiency to be commercialized worldwide. In addition, several vaccines have been developed against BLV that have been reported to be ineffective [17–20]. However, further studies are ongoing to develop new vaccines including the study from Archilla et al. [21] which has shown promising results but needs further investigation to assess the efficiency and biosafety of the vaccine.

**Fig. 1:**
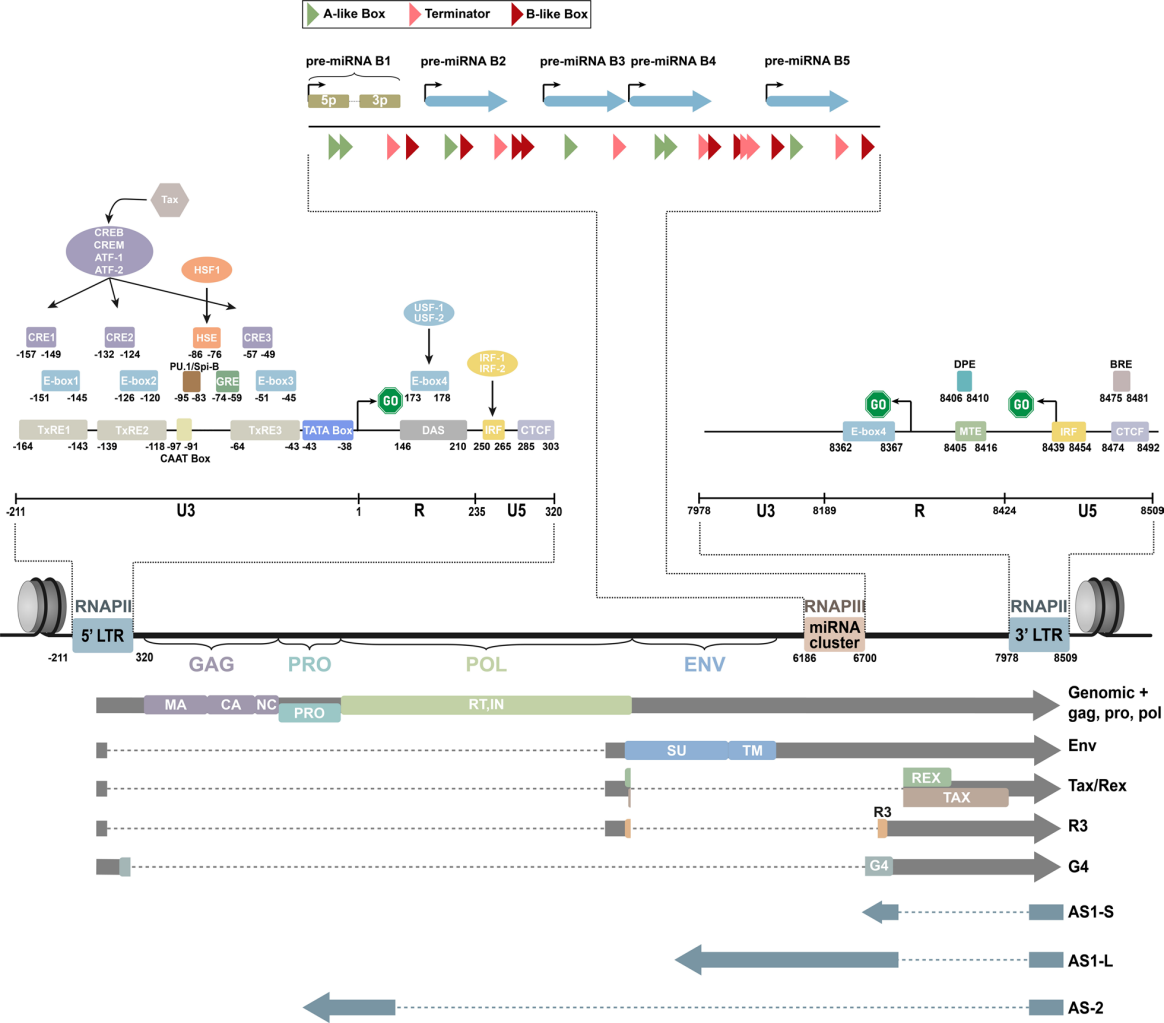
Schematic representation of the BLV genome. The BLV proviral genome is flanked by two identical Long Terminal Repeats (the 5ʹLTR and the 3ʹLTR). The 5ʹLTR contains the core RNAPII promoter elements (CAAT and TATA boxes) and associated *cis*-regulatory elements (see text) responsible for the expression of 5ʹ-capped unspliced (Genomic), single-spliced (Env, G4) or multi-spliced (Tax/Rex, R3) sense transcripts, each coding for different viral proteins. Through interactions with the cellular CREB, CREM, ATF-1 and ATF-2 transcription factors, the viral protein Tax transactivates the 5ʹLTR sense promoter activity. The miRNA region is composed of a cluster of 5 independent RNAPIII promoters, each containing characteristic type 2 RNAPIII promoter elements (A-like Box, B-like Box) and a terminator and is responsible for the constitutive expression of 5 pre-miRNAs (pre-miRNA-B1 to− B5) independently of Drosha. The pre-miRNAs are further processed by Dicer in 10 viral miRNAs (miR-B1-5p and 3p to miR-B5-5p and 3p). The 3’LTR exhibits a TATA-less antisense RNAPII promoter that operates thanks to a combination of core promoter elements (MTE, DPE, BRE) and different *cis-*regulatory elements (IRF, E-Box 4) and that is responsible for the expression of 3 single-spliced antisense transcripts (AS1-S, AS1-L, AS2) initiated from two major transcription start sites. Although the LTRs are identical, only *cis*-regulatory elements with known impact on sense or antisense promoter activity are represented in the 5ʹLTR or 3ʹLTR, respectively. Sequences of the described *cis-*regulatory elements can be found in Annex 1

As all retroviruses, BLV infects its target cells by retrotranscribing its single-stranded RNA genome into double-stranded DNA, integrating this DNA into the host cell genome, hijacking the cellular machinery to express the viral genes and complete the viral replication cycle. In this integrated form called provirus, the BLV genome is delimited by two identical DNA sequences, termed 5ʹ and 3ʹ Long Terminal Repeats (5ʹLTR and 3ʹLTR) (Fig. 1). The 5ʹLTR contains the promoter elements responsible for the RNA polymerase II (RNAPII)-dependent expression of the viral structural genes *gag*, *pro*, *pol* and *env* as well as of the accessory genes *R3*, *G4*, *Tax* and *Rex* necessary for efficient viral replication. However, rapidly after infection, the 5ʹLTR promoter activity is repressed through genetic and epigenetic mechanisms leading to the absence of viremia and the escape from the host immune system, which certainly contribute to tumoral development [22]. Despite this viral latency affecting the 5ʹLTR, BLV expresses 10 different miRNAs from a genomic region localized between the *Env* gene and the R3 coding region, through an RNA polymerase III (RNAPIII)-dependent process. Moreover, three antisense transcripts are expressed through an RNAPII antisense promoter activity located in the 3’LTR (Fig. 1).

Here, we will review the molecular mechanisms regulating each of the three BLV transcriptional activities, either through *cis*-regulatory elements or through epigenetic modifications: [1] the 5ʹLTR RNAPII-dependent sense transcription, [2] the RNAPIII-dependent miRNAs transcription and [3] the 3ʹLTR RNAPII-dependent antisense transcription. Additionally, we will describe the recently identified BLV miRNAs and antisense transcripts and summarize their implication in BLV-induced tumorigenesis.

## 5ʹLTR RNAPII-dependent sense transcription

In mammalian cells, gene expression depends on *cis*-regulatory sites including promoters, enhancers and silencers. The diverse patterns of gene expression are driven by the binding of transcription factors to promoters or enhancers which can be ubiquitous, cell-specific or induced by internal and external signals, leading to a multitude of gene expression profiles. Regarding BLV, viral gene expression is also regulated by the binding of cellular transcription factors and of the viral transactivator Tax to viral promoter and enhancer elements located in the 5ʹLTR. The 5ʹLTR (subdivided in 3 regions U3, R and U5) contains two canonical RNAPII core promoter elements, the CAAT and TATA boxes, responsible for transcriptional initiation at the U3/R junction of all viral sense transcripts [23, 24]. Functionally, different *cis*-regulatory elements bound by host cellular factors are responsible for a low basal transcriptional activity, while Tax is the unique viral protein required for the strong transactivation of the 5ʹLTR (Fig. 1). In addition to the cellular factors and the viral transactivator Tax, several epigenetic modifications influencing the chromatin state of the provirus also regulate the transcriptional activity of the 5ʹLTR.

### The *cis*-regulatory elements of the BLV 5ʹLTR

#### Tax-responsive elements

The most important sequences of the BLV 5ʹLTR are three 21 bp Tax-responsive elements (TxREs) located in the U3 region at positions nt− 164 to− 143 for TxRE1, nt− 139 to− 118 for TxRE2 and nt− 64 to − 43 for TxRE3 [25] (Fig. 1). Each TxRE is composed of an imperfectly conserved cyclic adenosine monophosphate (cAMP)-response element (CRE) (nt− 157 to− 149 for CRE1, nt− 132 to− 124 for CRE2, and nt− 57 to− 49 for CRE3) and an overlapping enhancer box (E-box) element (nt− 151 to− 145 for E-box1, nt− 126 to− 120 for E-box2, and nt− 51 to− 45 for E-box3). Importantly, all three TxREs have been demonstrated by deletion/mutation studies to be implicated in Tax-mediated transactivation of the 5ʹLTR [24, 26–29] that leads to a strong transcription of the viral genes, indicating that Tax is a key activator of BLV gene expression.

CRE-like motifs: A CRE motif is a palindromic octanucleotide consensus sequence 5ʹ-TGACGTCA-3ʹ bound by proteins of the CREB (CRE-binding proteins)/ATF (activating transcription factors) family, encoded by three homologous genes: *creb,* CRE modulator (*crem*) and activating transcription factor 1 (*atf-1)* [30]. The common feature between these transcription factors is the basic-region leucine zipper (bZIP) important for specific DNA binding and dimerization. These factors form homo—or heterodimers with CREB/ATF factors or with other factors containing a bZIP domain such as the activator protein-1 (AP-1), CCAAT/enhancer binding protein (C/EBP) or Maf family proteins to regulate gene expression [31].

To access CRE sequences and regulate gene expression, CREB needs to be phosphorylated on residue Ser133 [32]. The first kinase reported to phosphorylate CREB on Ser133 is the protein kinase A (PKA), an important effector of the cAMP signalling pathway. Later, other kinases have been demonstrated to phosphorylate CREB such as calcium/calmodulin-dependent kinases (CaMKs), mitogen-activated protein kinase (MAPK) and Akt [31]. The phosphorylation of CREB enables interaction with the co-activators CREB binding protein (CBP) and p300 that positively regulate gene expression through their histone acetyltransferase (HAT) activity and their interaction with the basal transcriptional machinery including TATA-box binding protein (TBP), TFIIB and RNAPII [31].

Concerning BLV, several laboratories have demonstrated by electrophoretic mobility shift assays (EMSAs) that the CRE-like motifs identified in the BLV 5’LTR are able to recruit the cellular transcription factors CREB [29, 33–35], CREM [36], ATF1 and ATF2 [35]. These in vitro binding studies were confirmed in vivo by chromatin immunoprecipitation (ChIP) assays in a B-lymphoid cell line isolated from a BLV-infected sheep at the tumoral stage, the YR2 cell line [36–38]. BLV latency in the YR2 cell line is due to two glutamic acid (E) to lysine (K) amino acid substitutions (E228K and E303K) in the viral protein Tax, impairing its transactivator role [39]. CREB/ATF have been shown to activate BLV transcription in basal condition (i.e. in the absence of Tax) by transient transfection experiments. Indeed, mutations in the CRE-like motifs markedly decrease the 5’LTR promoter activity with the mutation in the second CRE-like motif (CRE2) alone being sufficient to induce significant repression of the BLV promoter activity [38, 40]. Consistent with this, reverting the CRE-like motif to a perfect consensus sequence, thus favoring the binding of CREB/ATF proteins, results in an increased transcriptional activity of the 5’LTR [41]. Importantly, the three CRE-like motifs are necessary for Tax-mediated BLV transactivation [24, 26–29]. Indeed, the viral transactivator Tax does not bind directly DNA but interacts with the bZIP domain of the proteins of the CREB/ATF family, enhancing their binding affinity to the imperfect CRE elements and resulting in the high transactivation of BLV expression from the 5’LTR [42, 43]**.**

In contrast, not all members of the CREB/ATF family have been demonstrated to positively regulate the 5’LTR promoter activity, as some of them were shown to have no effect or even to negatively regulate Tax-mediated transactivation. For example, among the other isoforms of CREM, CREMτ activates the 5’LTR promoter activity, whereas it has no effect on its Tax-mediated transactivation [36]. However, co-transfection experiments of a 5’LTR reporter construct with expression vectors for CREB, Tax and CREMτ have demonstrated that CREMτ represses the synergistic activation of the BLV 5’LTR by CREB and Tax [36].

The activation of BLV gene expression by CREB/ATF family members seems to depend on the phosphorylation of the CREB/ATF factors and on the recruitment of their co-activators CBP/p300 to the BLV promoter as reported for HTLV-1 [44]. Co-transfection of CREB2 (also named ATF-4) with the kinases PKA [33] and CaMKIV [35] activates BLV transcription, while the absence of PKA inhibits Tax-mediated transactivation [33]. In addition, mutation of the Ser-117 in CREMτ, impairing its phosphorylation, leads to a decrease in the CREMτ-mediated activation of the 5ʹLTR promoter [36]. Moreover, recruitment of CBP to the BLV promoter has been shown in vivo by ChIP assays in PMA/ionomycin-stimulated YR2 cell line, and co-transfection of CBP with CREMτ induces a strong increase in the 5’LTR promoter activity compared to transfection of CREMτ alone, demonstrating a co-activation of the BLV promoter by CREMτ and CBP [36]. Mechanistically, the phorbol ester PMA and the calcium ionophore ionomycin activate the protein kinase C (PKC) and calcineurin, leading to the activation of several transcription factors such as NF-ƙB and nuclear factor of activated T-cells (NFAT), and finally causing BLV provirus reactivation [45].

These observations suggest that when a stimulus induces the cAMP pathway and/or an increase in calcium concentration, it activates the kinases PKA and/or CaMKIV, respectively, which in turn phosphorylate CREB/ATF factors. This allows their interaction with CBP and/or p300 which acetylate the histone tails (See ''Histone acetylation'' Sect), thereby activating the 5’LTR transcriptional activity. Importantly, it seems that phosphorylation of CREB/ATF factors is required for the transactivation of the 5ʹLTR by the BLV protein Tax.

E-boxes: In eukaryotic cells, E-box motifs are regulatory sequences with dual functions and able to bind numerous proteins. These proteins mainly contain a basic helix-loop-helix leucine-zipper domain and can act depending on their binding partners either as transcriptional activators (such as enhancer-binding protein 4 (AP-4), upstream stimulatory factors 1 and 2 (USF1 and USF2)) or as transcriptional repressors (such as MYC associated factor X (Max), MAX dimerization protein (Mad)) [46, 47].

In the context of BLV, these motifs have been initially described as binding sites for the cellular factor AP-4, as the two first E-box motifs contain the consensus sequence 5’-CAGCTG-3’ corresponding to the AP-4 binding motif [25, 48]. However, to date, the identity of the factors binding to the E-box1, 2 and 3 remains to be established. In vitro supershift assays using antibodies targeting potential candidates such as AP-4 [41], but also USF-1, USF-2, Max, Mad1 to Mad4 and activated B-cell factor 1 (ABF-1) have revealed that these factors do not bind to the E-box motifs [40]. Concerning the functional effects of the E-boxes on the 5’LTR promoter activity, conflicting results have been reported, either in basal or Tax-transactivated conditions. In basal condition, transient transfection experiments of a 5’LTR-reporter construct containing the E-boxes 1 or 2 mutated individually have shown an activating or inhibitory role depending on the transfected cell line [25]. However, no molecular evidence explaining these results has been given [25]**.** On the contrary, the combined mutations of the 3 E-boxes result in a 2 to fourfold increase in the 5’LTR promoter activity when the corresponding reporter construct was transfected in two different cell lines (Raji or D17) [40, 41]. Molecularly, this repressive role of the E-boxes has been attributed to a steric hindrance occurring between the potential proteins bound to the E-boxes and the overlapping CRE regions, as demonstrated by EMSAs [40]. In Tax-transactivated condition, the E-box motifs can either enhance or repress Tax activity in reporter assays, depending on the transfected cell line [25].

These results suggest a complex mechanism of BLV promoter regulation depending on the cell state, the recruitment of factors and the presence of the viral transactivator Tax.

#### PU-box

Our laboratory has identified a PU-box in the U3 region of the BLV 5ʹLTR located between the positions nt− 95 to− 83 and centered on a purine-rich motif GGAA [49]. PU.1 and Spi-B, both B-cell- and macrophage-specific members of the ETS transcription factor family [50], have been demonstrated to specifically interact with the PU-box by gel shift assays. Moreover, PU.1 recruitment to the 5’LTR has been demonstrated in vivo by ChIP assays in the YR2 cell line and is increased by treatment of the cells with a combination of phorbol-12-myristate 13-acetate (PMA) and ionomycin (PMA/ionomycin), a potent activator of BLV gene expression [51]. Interestingly, our laboratory has also shown that ectopic expression of PU.1 and Spi-B significantly increases transcription from the BLV promoter in absence of Tax [49]. Consistent with this, a 2 bp mutation in the PU-box reduces the BLV 5’LTR promoter activity as well as its activation following PMA/ionomycin treatment [40, 49, 51]. However, the same mutation did not affect the responsiveness of the BLV promoter to the viral transactivator Tax, indicating that PU.1 and Spi-B positively regulate the basal transcription but are not required for Tax-mediated transactivation of the BLV promoter. PU.1 is well known for its key implication in the determination of the identity of multiple cell lineages of the immune system such as myeloid, dendritic and B cells [52, 53] by acting as a “pioneer” transcription factor in opening closed chromatin [54], thereby leading to the recruitment of other transcription factors [55]. With its specificity for B cells and its “pioneer” action, PU.1 could be a key factor in BLV transcription by providing access to the BLV promoter to other transcription factors, such as CREB/ATF, USF, and IRF, and leading to the expression of Tax and the transactivation of the BLV promoter. Importantly, the PU.1/Spi-B binding site located in the BLV 5’LTR constitutes the only *cis*-regulatory element recognized by transcription factors whose expression is restricted to macrophages and B cells and could thus participate to the BLV tropism for B cells.

Before the identification of the PU.1 binding site, Brooks et *al*. had suggested the presence of a ƙB binding site at a position between nt− 118 and nt− 70 [56]. However, our laboratory could not demonstrate the binding of NF-ƙB by both supershift and competition experiments using nuclear extracts and the same DNA sequence used by Brooks et *al*. [49, 56].

#### Heat shock element (HSE)

In mammals, following heat shock, the transcription factor heat shock factor 1 (HSF1) drives the transcriptional responses by binding to HSE [57]. HSF1 induces the expression of genes of the heat shock response corresponding mainly to chaperones. In the case of BLV, Hachiya et *al.* [58] have identified a conserved HSE sequence (5ʹ-TTTCCCGAAA-3ʹ) in the U3 region of the BLV 5ʹLTR between the positions nt− 86 to− 76, overlapping the end of the PU-box. By luciferase reporter assays in feline CC81 cells, these authors have shown the basal activation of the BLV LTR by HSF1. Moreover, HSF1 has also been demonstrated to increase Tax-mediated transactivation of the BLV LTR in these transient transfection experiments. The deletion of the HSE sequence in the BLV LTR and the use of a HSF1 mutant missing the DNA-binding domain impaired the BLV LTR-activation by HSF1 [58]. Therefore, the positive regulation of the BLV LTR by the cellular transcription factor HSF1 is mediated through binding to the HSE sequence.

#### GRE element

In 1979, Bloom et *al*. have reported that B lymphocytes from BLV-infected cows are sensitive to glucocorticoids in vitro and in vivo [59]. Later, Niermann and Buehring have focused on the effects of mammotropic hormones on BLV gene expression and tested five different hormones: 17β-estradiol, progesterone, insulin, prolactin, and dexamethasone [60]. Among these five hormones, the glucocorticoid dexamethasone possesses the most stimulatory effect which is increased by the addition of insulin and prolactin. However, the effect of dexamethasone requires the presence of BLV Tax and the glucocorticoid receptors [60]. One year later, the same team has identified a putative glucocorticoid response element (GRE) localized upstream of the CRE3 which binds purified glucocorticoid receptor protein [25]. The basal activity of the BLV LTR is significantly impacted by mutations in the GRE site in transient transfection assays [25, 40, 41]. However, more recently, Jaworski et *al.* have assessed the effect of dexamethasone on BLV infection in cattle chronically infected with BLV [61]. They have reported a spontaneous BLV reactivation in the cattle that was independent of dexamethasone treatment, suggesting the involvement of other important stimuli that still need to be identified. Overall, further studies are required to better understand the roles of the GRE element located in the BLV LTR and of glucocorticoids in BLV transcriptional regulation and pathogenesis.

#### Downstream activator sequence (DAS)

Derse and Casey have described a 250 bp-long element in the R/U5 region that strongly increases the activity of the BLV promoter independently of the viral transactivator Tax [23]. The section of the 250 bp long element containing the regulatory part was determined by deletion mutants of the U5 region in a transient expression system [62]. It corresponds to a 64 bp fragment called DAS [62] and located between positions nt + 146 to + 210 in the U5 region of the BLV 5’LTR. DAS contains two independent overlapping elements named DAS1 for the proximal element and DAS2 for the distal element that are each composed of three boxes required to upregulate the BLV promoter activity [62]. The strong upregulating activity of DAS suggests that it might be an enhancer of the BLV promoter independent of viral factors.

#### E-box 4

Besides the E-boxes located in the three TxREs, our laboratory has characterized an E-box motif 5’-CAGGTG-3’ located in the R region (nt + 173 to + 178) named E-box4 [63], inside the DAS region previously described. Unlike the other E-boxes, the specific binding of the basic helix-loop-helix proteins USF1 and USF2 to the E-box4 motif was demonstrated by electrophoretic mobility shift assays (EMSAs) [63] and further confirmed in vivo by ChIP-qPCR assays [51].

Functionally, the E-box4 motif is described as an activator of both basal and Tax-transactivated 5’LTR promoter activities, as demonstrated by transient transfection assays [40, 63]. Moreover, the E-box4 is important for the responsiveness of the BLV promoter to PMA/ionomycin treatment. Indeed, the treatment efficiency is reduced by mutating the E-box4, while it increases the binding of USF-1 and USF-2 through phosphorylation by an uncharacterized mechanism [51]. Additionally, the ectopic expression of USF1 and USF2 induces a stimulatory effect on the BLV promoter which is in part dependent on the E-box4 motif [63]. Interestingly, the effect of USF1 and USF2 is also impacted by mutations in the three other E-box motifs, suggesting that USF factors might also bind to those sequences [63]. Furthermore, the mutations in the four E-box motifs do not completely abrogate the transactivation of the BLV promoter by USF1 and USF2a, suggesting that they might interact with other *cis*-elements located in the BLV promoter region [63]. Thus, USF positively regulates BLV transcription through the four E-boxes and might also directly or indirectly interact with other *cis*-regulatory elements.

#### IRF binding site

In the U5 region of the BLV LTR, a transcriptional enhancer that contains a binding site for an interferon regulatory factor (IRF) was discovered between position nt + 250 to nt + 265 [64]. Our laboratory has shown that the factors IRF1 and IRF2 bind in vitro to the IRF binding site by gel shift assays [64]. Moreover, deletions and mutations in the IRF binding site decrease the basal activity of the BLV promoter in transient transfection assays [40, 64]. These results suggest that IRF-1 and IRF-2 could be involved in the initiation of BLV gene expression. However, the effects of IRF-1 and IRF-2 on BLV gene expression in transactivated conditions have not been reported, therefore a role of IRF-1 and IRF-2 after initiation of the transcription should not be excluded.

#### CTCF binding site

The CCCTC binding factor (CTCF) is a cellular factor with multiple transcriptional and epigenetic regulatory roles. CTCF uses different combinations of its 11 zinc finger domains to directly bind DNA regions harboring a conserved 15 bp core palindromic motif [65]. Mostly known for its importance in the regulation of the three-dimensional structure of the chromatin by forming chromatin loops between two CTCF binding sites [66–68], CTCF may also directly act as a transcriptional activator or repressor depending on its interacting partners [69]. Recently, we have shown by ChIP-qPCR in BLV-infected ovine cell lines and ovine PBMCs that CTCF binds in vivo to three regions of the BLV genome: at the U5 region of both the 5’LTR (nt + 285 to + 303) and the 3’LTR (nt + 8474 to + 8492), and to a lesser extent to the region coding for the second exon of Tax/Rex (nt + 7304 to + 7322) [70]. Transient transfection assays of a reporter construct containing the BLV 5’LTR wild-type or mutated for the CTCF binding site cloned upstream of the luciferase reporter gene demonstrated that CTCF acts as a repressor of the 5ʹLTR promoter activity in basal conditions, yet through an uncharacterized molecular mechanism. Moreover, besides CTCF-mediated repression by direct binding to the 5ʹLTR, the CTCF binding site located at the second exon of Tax/Rex might contribute to the repression of the 5ʹLTR by defining a histone modification profile and preventing the spread of activating histone marks towards the 5ʹLTR [70].

## Impact of the chromatin environment on the BLV sense transcription

Transcriptional activity and establishment of viral latency are largely influenced by the genomic environment surrounding the provirus. Retroviruses integrate the host cellular genome, allowing the use of the transcriptional machinery but also subjecting the provirus to all the mechanisms regulating cellular gene expression.

In the nucleus of eukaryotic cells, DNA is packaged in a structural and functional repeating unit called the nucleosome composed of a DNA segment of 146 bp wrapped around a central core octamer of histone proteins. This octamer is composed of two molecules of each canonical histone proteins H2A, H2B, H3, H4. Each nucleosome is separated from the next one by a linker DNA, forming a polynucleosome fiber further stabilized by the histone H1 binding to the linker DNA close to the core histone octamer. Each histone contains a N-terminal domain that protrudes out of the nucleosome structure and that is subjected to several post-translational modifications [71]. Besides its role in compacting the DNA in the nucleus, it is now clear that chromatin structure regulates all the aspects related to DNA, such as recombination, DNA repair, replication, and importantly, regulation of gene expression, mainly by modulating the accessibility of DNA for regulatory transcription factors (reviewed in [72, 73]). Chromatin can be in a condensed structure characterized by regularly positioned nucleosomes, called heterochromatin, generally associated with transcriptionally inactive regions of the genome. In contrast, nucleosomes may be organized in a more dynamically regulated and loose structure associated with transcriptionally active regions of the genome, called euchromatin [74]. In this context, the position of the nucleosomes relative to a promoter has been shown to directly regulate its transcriptional activity [75]. In retroviruses, the nucleosomal organization surrounding the viral promoter has been shown to play an important role in HIV-1 [76] and BLV latency [51]. Similar to what is observed in HIV-1, our laboratory has demonstrated by indirect end-labelling experiments in the context of BLV-latently infected cell lines and ovine PBMCs that a nucleosome is positioned in the R region of the BLV 5ʹLTR, contributing to maintain a repressive closed chromatin structure [51]. Moreover, we have demonstrated that treatment of the latently-infected ovine cell line YR2 with a combination of PMA/ionomycin induces a disruption of the nucleosome positioning accompanied by BLV reactivation [51].

In addition to changes in nucleosome positioning, the core histones and particularly their unstructured N-terminal tails, are subjected to various reversible post-translational modifications also regulating the expression of surrounding genes. These modifications include acetylation, methylation phosphorylation, SUMOylation, ADP-ribosylation, ubiquitylation and other less represented modifications (reviewed in [77–79]) resulting in the modulation of gene expression by two mechanisms: [1] by directly altering the chromatin packing through changes in electrostatic charges altering DNA-histone contacts or inter-nucleosomal contacts, thus controlling the accessibility of transcription factors to DNA; [2] by displaying specific histone modifications patterns recognized by chromatin-associated proteins, with different downstream effector functions [80]. Remarkably, the association of different histone modifications can define the transcriptional state of different genomic locations such as promoters, enhancers or gene bodies and can be associated to activation or repression of transcription. Among the different post-translational modifications, lysine acetylation, lysine methylation and arginine methylation are the best characterized.

### Histone acetylation

Histone acetylation is a reversible post-translational modification widely associated to transcriptional activation and consists in the transfer of an acetyl group onto the amino termini group of a lysine residue [79]. By doing so, the natural positive charge of the lysine is neutralized, which in turn weakens the DNA-histone interaction and favors the accessibility of transcription factors to DNA [81]. Moreover, transcriptional activation is also favored by specific recognition of acetylated lysine residues by bromodomain (BRD)-containing proteins, such as remodeling complexes and transcription factors [82, 83]**.** For example, the BRD-containing protein BRD4 favors transcription by binding to acetylated histones H3 or H4 and by recruiting the positive transcription elongation factor b (P-TEFb), playing a critical role in transcriptional elongation [84, 85]. Lysine acetylation is regulated by two groups of enzymes with opposing functions: the histone acetyltransferases (HATs) and the histone deacetylases (HDACs). The HATs (subdivided in four families: GNAT, MYST, CBP/p300 and others) [86, 87] catalyze the transfer of the acetyl group from an acetyl-CoA molecule mainly to the amino termini of lysines [88]. On the contrary, HDACs (subdivided in four classes based on sequence similarities: HDACI, II, III, IV) catalyze the removal of the acetyl group [87, 89].

Several studies have demonstrated the involvement of histone acetylation in the transcriptional regulation of BLV. First, the group of Richard Kettmann in collaboration with our laboratory has shown that HDAC inhibitors (HDACi) such as trichostatin A (TSA) and trapoxin (TPX) induce a marked increase of the BLV promoter activity in integrated reporter constructs in the D17 cell line [90]. In addition, TSA induces a strong BLV gene expression in peripheral blood mononuclear cells (PBMCs) from sheep and cattle that is comparable to the induction observed using PMA/ionomycin which is the most potent combination to activate BLV gene expression [90]. Another HDACi, the antiepileptic drug valproate, was demonstrated to activate LTR-driven gene expression in transient transfection experiments and in ex vivo cultures of PBMCs isolated from a BLV-infected sheep [91].

Our laboratory has confirmed the effects of TSA and valproate on the BLV promoter and has shown that another HDACi, sodium butyrate (NaB), exerts similar activations of the BLV promoter activity in transient transfection assays and in the context of an integrated provirus [40]. Moreover, we established a correlation between the degree of histone acetylation at the BLV 5ʹLTR and the transcriptional activation of the viral promoter by TSA. Indeed, we reported an increase in histone H4 acetylation at the BLV promoter by ChIP assays following treatment with the HDACi TSA. In addition, ectopic expression of each HDACs 1 to 5 induces a significant downregulation of LTR-driven gene expression in transient transfection assays [40].

The effects of HDACis are in part linked to the 3 E-box motifs overlapping the CRE-like motifs located in the TxREs. Indeed, mutations in the E-box motifs decrease the responsiveness of the BLV promoter to TSA without completely abolishing this responsiveness [40]. The observed repressing role of the E-boxes could be linked to the recruitment of HDACs to the BLV promoter. In addition, the in vitro binding of CREB/ATF is increased in gel shift assays using nuclear extracts from PBMCs and chronically BLV-infected cells treated with TSA [40, 92, 93]. In agreement, the in vivo recruitment of CREB to the BLV promoter is increased by TSA treatment of the YR2 BLV-infected cell line [92].

The group of Anne Van den Broeke has demonstrated the in vivo recruitment of HDAC1 and the known co-repressor mSin3A to the BLV promoter in the BLV-infected L267 cell line [93]. Following treatment of L267 cells with a combination of the DNA demethylating agent 5-azacytidine (5-azaC) and TSA, the recruitment of HDAC1/mSin3A to the 5’LTR was decreased and correlates with an increase in histone H3 and H4 acetylation, as well as with reactivation of BLV gene expression in the latent L267 cell line. Accordingly, the recruitment of HDAC1 to the BLV promoter in vivo was confirmed by our laboratory in another BLV-latently infected cell line YR2 [51] and we have shown that reactivation of BLV gene expression by PMA/ionomycin induces a decrease in HDAC1 recruitment which is inversely correlated with histone H4 acetylation levels.

In line with the activating effects of HDACis, a synergism between Tax and HDACis (TSA and NaBut) was observed, which was dependent on the CRE-like motifs in the U3 region of the 5’LTR [92]. Indeed, mutations in the 3 CRE motifs or ectopic overexpression of a negative dominant form of CREB abrogates the strong transactivation of the BLV promoter by Tax + TSA or Tax + NaBut in luciferase reporter assays [92].

Factors containing an intrinsic HAT activity such as CBP/p300 have been shown to be recruited to the BLV promoter in vivo by ChIP assays using chromatin from YR2 cells treated with PMA + ionomycin [36]. In transient transfection systems, CBP has been demonstrated to activate BLV basal transcription and to be involved in the activation of the BLV 5ʹLTR by CREMτ [36]. The study suggests that CBP is recruited to the BLV promoter by CREMτ.

In conclusion, modulation of histone acetylation at the BLV promoter is important for BLV transcriptional regulation.

### Histone lysine methylation

Unlike histone acetylation which generally favors transcriptional activity, histone methylation has positive or negative effects on gene expression depending on the specific residues that are modified, the degree and pattern of modifications and the genomic localization of the modified histones [94]. In histones, the methylation process involves the attachment of a methyl group on the amino acid side chain and/or at the amino termini of lysine or arginine residues. Due to the multiple nitrogen atoms subjected to methylation in arginine residues, arginine methylation is complex and its role in gene expression is poorly understood. On the contrary, methylation of lysines occurs at the unique terminal amino group, and can exist as a mono-, di- or tri-methylated lysines. Although all the core histones may be methylated, histone H3 is principally methylated and typical marks can be distinguished: mono-, di-, or tri-methylated H3K4, H3K36 and H3K79 are typically characteristic of transcriptionally active regions, while methylated H3K9 and H3K27 are typically gene-repressive [94]**.** For example, H3K4me3 [95] is a characteristic mark of active promoters, and H3K4me1 is an activating mark of enhancers [96]. On the other hand, H3K9me2 is more frequently found at silent genes and H3K9me3 is characteristic of heterochromatin.

In opposition to lysine acetylation, methylation does not change the electronic charge of the side chain. Therefore, lysine methylation impacts gene expression mainly through effector proteins containing methyl-lysine-binding domains, such as the PHD, chromo, MBT domains and others [97]. As an example, the chromodomain-containing protein HP-1 specifically recognizes H3K9me3, which in turn induces the recruitment of the histone methyltransferase SUV39H1, which propagates the heterochromatin structure by additional H3K9me3 modifications. Histone methylation is a dynamic process regulated by different groups of proteins known as histone methyltransferases (HMTs), such as SUV39H1, G9a, EZH2, and histone demethylases (HDMTs), such as LSD1 and JmjC domain-containing enzymes catalyzing the addition or the removal of the methyl group respectively [97].

Less is known about the implication of histone methylation in BLV 5’LTR transcriptional regulation. In the latently-infected L267 cell line, the 5’LTR is associated with low levels of activating H3K4 methylation marks, in accordance with 5’LTR transcriptional repression [70, 93, 98]. However, the group of Anne Van den Broeke has described by ChIP assays that reactivation of the cells with the combination of TSA and 5-azaC induces an increase in H3K4 methylation marks [93]. The same methylation changes have been reported following viral activation with the PMA + ionomycin combined treatment of the YR2 cell line [51], suggesting that histone methylation also contributes to the regulation of BLV promoter activity.

### DNA methylation

Another epigenetic mark known to be involved in the transcriptional regulation of BLV is DNA methylation. DNA methylation mainly occurs on cytosines located within CpG dinucleotides in mammalian cells. This modification is written par DNA methyltransferases (DNMTs) that catalyze the transfer of a methyl group from S-adenyl methionine to the fifth carbon of a cytosine, forming 5-methylcytosine [99]. During DNA replication, the DNA methylation profile is maintained by DNMT1 and the de novo methylation is due to DNMT3a and DNMT3b [100]. However, it is now admitted that DNMT1 can also catalyze de novo DNA methylation [101] and that DMNT3a and DNMT3b can also contribute to the maintenance of DNA methylation patterns [102]. Generally, DNA methylation in transcriptional regulatory regions is associated with gene silencing, either by directly blocking the binding of transcription factors to their recognition sequences or by indirectly preventing transcription factors from accessing their target sites through the attachment of methylcytosines-recognizing proteins. These latter proteins interact with and recruit epigenetic enzymes, such as HDACs and HMTs, thereby resulting in the formation of a closed repressive chromatin structure [103–105].

Concerning BLV, our laboratory has determined the DNA methylation status of the 5’LTR promoter region in two lymphoma-derived BLV latently-infected ovine B cell lines (the L267 cell line and the YR2 cell line) by bisulfite sequencing method [38]. The L267 cell line is composed of a fully replication-competent provirus [93] and possesses a hypermethylated promoter [38], whereas the YR2 cell line possesses a hypomethylated promoter [38] and is defined by a single monoclonally-integrated silent provirus, in which infectious potential is impaired by two mutations in the viral transactivator Tax [106, 107]_._ L267 is thought to represent a true latency state, while YR2 represents a mutant defective latency. Moreover, the reactivation of the L267 cell line by transduction of Tax (L267 Tax cell line) induces global demethylation of the 5ʹLTR [38, 93]. In addition, only the latent promoter in the 5ʹLTR is hypermethylated in the U3 and R regions, while the active promoter at the 3ʹLTR is fully hypomethylated. These observations suggest a correlation between the DNA methylation at the 5’LTR and transcriptional repression of the BLV promoter.

The methylation status observed in the L267 Tax cell line suggests preferential demethylation of the two CRE-like motifs of the TxRE1 and TxRE2 [38]. By gel shift assays, the binding of CREB/ATF proteins to the TxRE1 and TxRE2 was demonstrated to be impaired by DNA methylation of a single CpG dinucleotide in vitro. In agreement, their recruitment to the BLV promoter in vivo occurs in the YR2 cell line and not in the L267 cell line possessing a hypermethylated promoter.

In vitro methylation of the BLV promoter induces a decrease in basal [38, 108] and transactivated transcription [108] in transient transfection assays. The activity of the in vitro methylated BLV promoter is increased following treatment with different DNA demethylating agents (procaine, procainamide, 5-aza-2ʹ-deoxycytidine (5-azadC), and zebularine) [38]. Another study has shown the reactivation of BLV latently-infected cells following the treatment with a combination of TSA and 5-azaC [93]. A synergism between Tax and the DNA methylating agents, 5-azadC and procaine, was demonstrated and is dependent on the three CRE-like motifs of the TxREs in mutant studies [38]. Moreover, ectopic expression of DNMT1 and DNMT3A, not DNMT3B, decreases the transcription of the BLV promoter [38]. Interestingly, the expression of the DNMTs is downregulated by the viral transactivator Tax.

The methylation status of the BLV promoter in vivo, in the context of the natural host, has been controversial. Kashmiri et *al.* have shown the hypermethylation of the proviral promoter in both BLV-induced tumor cells and in PBMCs from BLV-infected cattle with lymphosarcoma using methylation-sensitive restriction enzymes [109]. However, Tajima et *al.* have shown, by bisulfite sequencing, the absence or poor DNA methylation of the 5ʹLTR in PBMCs of cows at different stages of the disease and of a sheep experimentally infected by BLV [108]. These conflicting results might be explained by the fact that integrated provirus in tumoral cells can be completely silenced by genetic alteration of its sequence and thus the cells studied might be silenced by mutations or deletions in the provirus [108].

Overall, these results suggest that the DNA methylation of the BLV 5ʹLTR plays an important role in the transcriptional repression of the viral promoter in the context of a fully replication-competent integrated provirus.

These studies demonstrate the importance of the chromatin environment in the transcriptional regulation of the BLV promoter and its pathogenesis. Moreover, drugs targeting mechanisms of epigenetic regulation are promising in terms of therapeutic approaches.

## RNAPIII-dependent expression of BLV miRNAs

MicroRNAs (miRNAs) are a class of small single-stranded noncoding RNAs of approximately 22 nt in length that play important roles in gene expression regulation at the post-transcriptional level [110, 111]. First discovered in eukaryotes, it is now clear that viruses, mainly with DNA genomes but also RNA viruses, express viral miRNAs which play important roles in viral replication cycle and disease development [112–114]. Canonically, eukaryotic and viral miRNAs are transcribed from either the protein-coding region or the noncoding region of the genome by RNAPII to generate long hairpin structured primary miRNA transcripts (pri-miRNAs) [112, 115]. Pri-miRNAs are then processed by the nuclear RNaseIII Drosha to generate single hairpin called precursor miRNA (pre-miRNA). After their export in the cytoplasm by Exportin 5, the pre-miRNA is cleaved by Dicer to generate a double-stranded miRNA intermediate. Then, the strand with the most thermodynamically stable 5’-end (called mature miRNA) is incorporated in the RNA-induced silencing complex (RISC) composed of the Ago protein. The RISC complex is then guided by sequence complementarity of the miRNA to the target RNA, where it promotes its translation inhibition and/or its degradation. Non-canonical miRNA biogenesis pathways have also been characterized and can be classified in Drosha- or Dicer-independent pathways [115, 116].

Concerning BLV, the groups of Christopher Sullivan [117] and Anne Van den Broeke [118] have simultaneously identified that a genomic region of ~ 600 nt expresses a cluster of 10 miRNAs (BLV-miR-B1-3p/5p to BLV-miR-B5-3p/5p) (Fig. 1) through a non-canonical process by the use of bioinformatic prediction tools and high-throughput sequencing of small RNAs. Indeed, the BLV miRNAs are expressed through five independent type II RNAPIII promoter units, by a Drosha-independent mechanism [98, 117, 118]. Type II RNAPIII promoters, responsible for tRNAs expression, typically contain two intragenic regulatory elements (the A box and the B box) followed by a stretch of 4–6 polyA on the template DNA strand as terminator signal [119]. Unlike this typical organization, each BLV miRNA promoter is constituted of one or two intragenic A-box separated from a B-box by the terminator signal and individual mutation in each of these elements has revealed their importance for proper pre-miRNA expression [120]. Through this RNAPIII-dependent expression, BLV miRNAs are individually embedded in a 5ʹ triphosphorylated pre-miRNA structure with 5ʹ and 3ʹ extremities defined by the transcription start and end sites, respectively [120]. Moreover, reporter assays with the miRNA cluster cloned in the 3ʹUTR of the luciferase gene has revealed that longer RNAPII transcripts are not processed neither by Drosha, nor by other Drosha-independent mechanisms [120]. In other words, these results demonstrate that longer viral transcripts containing the miRNA cluster (such as the genomic, *Gag* or *Env* transcripts) are not cleaved by Drosha avoiding a potential detrimental effect on BLV replication due to degradation of viral transcripts [117, 118]. The 5ʹ triphosphorylated pre-miRNAs are then exported in the cytoplasm and processed by Dicer, prior or concurrently being dephosphorylated by an unknown triphosphatase activity [120]. Then, one strand of the miRNA duplex associates with an Argonaute (Ago) protein to form the RISC complex and mediates gene silencing.

### Roles of the BLV miRNAs

Previous reports have demonstrated that the region now called miRNA cluster is dispensable for BLV infectivity [121, 122]. However, an accumulating number of reports now demonstrates that the miRNA cluster is important for viral replication and/or oncogenesis [117, 118, 123–126]. Indeed, sequence conservation analyzes from different BLV strains have revealed a high conservation rate of the miRNA cluster, particularly the promoter elements (A-box, B-box and terminator) and the seed region (nt 2–7 of the mature miRNA) that targets RNA [118, 127]. Moreover, RNA-sequencing assays performed on primary ovine and bovine pre-leukemic and malignant B cells have revealed the high expression of BLV miRNAs, which can reach up to 40% of total cellular miRNAs [117, 118, 123, 124]. Also, BLV miRNAs are detected at high levels in the serum of infected animals, pointing out potential paracrine roles [125, 128]. All these elements suggest that BLV miRNAs might play important roles in retroviral cycle and/or disease development. Indeed, sheep experimentally infected with BLV provirus deleted for the miRNA cluster (∆miRNA) have shown a lack of tumorigenesis compared to the wild-type sheep, which have developed tumors ~ 22 months post-infection [125, 126]. Consistently, RNA-seq analysis has revealed the deregulation of several biological pathways such as inflammatory response, immunity, cell signaling and proliferation, as demonstrated by the higher proliferation rate of B lymphocytes in BLV wild-type infected animals compared to the BLV ∆miRNA-infected animals [126]. In the bovine species, evaluation of the role of miRNA in tumoral development would take decades, due to the long latency period and the low rate of oncogenesis (5%) [1]. However, proviral load is a good prediction marker of pathogenesis [126] and cattle experimentally infected with BLV ∆miRNA have shown a lower proviral load than animals infected with the wild-type BLV, suggesting that tumoral development will be decreased in BLV ∆miRNA-infected animals [125]. In this study and similar to what is observed in sheep, RNA-seq from PBMCs extracted from BLV wild-type- or ∆miRNA-infected calves have revealed that BLV miRNAs modulate the expression of genes involved in immune response, cell signaling, apoptosis and oncogenesis [125]. Functional in vitro reporter assays have confirmed that some of these downregulated genes such as *GZMA*, *FOS* and *PPT1* are directly targeted by one BLV mature miRNA, the blv-miR-B4-3p [125]. Blv-miR-B4-3p shares seed sequence similarities with the host miRNA miR-29a, which is known to downregulate the expression of two tumor suppressor genes, the genes coding for the HMG-box transcription factor 1 (HBP1) [129] and for the peroxidasin homolog (PXDN) [130], in B-cell tumors. In vitro reporter assays have shown that these two genes were also directly downregulated by blv-miR-B4-3p [131]; however, these results were not confirmed in bovines experimentally infected with BLV [125]. Nonetheless, in a recent study analyzing the expression of *hbp1* and *pxdn* by RT-qPCR in the context of bovines naturally infected with BLV, Petersen and colleagues have observed a downregulation of *pxdn* compared to uninfected animals [132]. Apart for blv-miR-B4-3p, detailed analysis of the targets of each individual BLV mature miRNA has not been performed but could provide important insights into the role of BLV-induced tumorigenesis.

### Regulation of miRNA expression

In contrast to the extensively analyzed regulation of RNAPII transcription, RNAPIII transcriptional regulation is considerably less understood in detail, although generally considered as simpler. Unlike RNAPII genes, RNAPIII activity does not seem to be regulated by transcription factors binding to DNA sequences upstream or downstream from the promoters elements. However, recruitment of the transcription factors essential for RNAPIII assembly is directly impacted by several factors, such as p53, Rb, Maf1 or Myc, or by epigenetic mechanisms such as histone acetylation, histone methylation and DNA CpG methylation [133, 134].

Although most BLV miRNAs are highly expressed in both pre-leukemic and malignant BLV-infected B cells [118] and correlate with proviral load [123], little is known about their expression throughout the development of the disease, starting from the infection. Only one report in the context of BLV-infected ovine B cell lines has demonstrated that the miRNA cluster is associated with the presence of activating epigenetic marks, as well as the absence of repressive histone marks and methylated CpG dinucleotides, in agreement with the high expression of the BLV miRNAs [98].

## 3ʹLTR RNAPII-dependent antisense transcription

Transcription from the 3’LTR in opposite orientation of the transcription initiated from the 5’LTR promoter, hereafter called antisense transcription, is now considered as a common theme amongst retroviruses [135] such as HIV-1 [136], HTLV-1 [44] and more recently BLV [137]. Indeed, by RNA-seq analysis of total RNA extracted from BLV-infected ovine and bovine samples, Durkin et al. [137] have identified antisense transcripts originating from the 3ʹLTR, despite the latency affecting the 5ʹLTR. More precisely, three antisense spliced transcripts have been identified (Fig. 1). The first and most abundant antisense transcript (AS1-S) is a ~ 600 bp spliced transcript that can be extended by alternative polyadenylation in a second longer transcript of ~ 2200 bp overlapping the miRNA cluster, called AS1-L [137]. For both AS1-S and AS1-L transcripts, canonical AAUAAA polyadenylation signal sequence (PAS) is found. However, there is no downstream classical GU-rich consensus sequence. Moreover, polyadenylation is not confined to a single region but located across various locations in a region of ~ 60 bp. The third antisense transcript (AS2) is a ~ 400 bp transcript generated by alternative splicing that does not show, in the majority of the cases, evidence of polyA tail. Instead, AS2 uses integration site-dependent host splice acceptor sites to form virus-host fusion transcripts [137].

### 3ʹLTR RNAPII antisense promoter

Unlike the BLV 5ʹLTR promoter, the 3’LTR is a TATA-less promoter that initiates transcription at multiple transcription start sites [137]. Indeed, 5ʹ rapid amplification of cDNA ends (5ʹRACE) assays followed by high-throughput sequencing has identified several transcription start sites: one in the R region (nt + 8380) and one in the U5 region (nt + 8438) of the 3ʹLTR, responsible for the initiation of ~ 25% and ~ 45% of antisense transcripts, respectively [137]. By in silico search of core promoter motifs and mutagenesis of reporter constructs, our laboratory has functionally identified a combination of core promoter elements, a TFIIB-Recognition Element (BRE) (GGGCGCC) and an overlapping Motif Ten Element (MTE) (CAAGCCAGACGC)/Downstream Promoter Element (DPE) (AGACG) as highly important for BLV antisense transcription (Fig. 1) [98]. Other less critical *cis*-regulatory elements have been identified through similar approaches of reporter constructs, such as the previously identified IRF and E-box4 motifs located in the U5 and R regions, respectively [137]. Also, CTCF seems to positively regulate the 3ʹLTR antisense promoter activity, as abolition of its recruitment to the binding site located at the U5 region drastically reduces BLV antisense transcriptional activity [70]. On the contrary, *cis*-regulatory elements negatively regulating antisense transcription also exist, as deletion of the U3 region in the 3ʹLTR results in an increase in antisense promoter activity. However, the precise contribution of each regulatory element located in the U3 region is still unknown [137]. Finally, the role of the viral transactivator Tax protein on 3ʹLTR antisense promoter activity is still unclear. Indeed, Tax has been shown to transactivate antisense transcription in the context of a 3ʹLTR reporter construct transfected in the human B cell line Raji [98], while the opposite effect has been observed by another group when a similar reporter construct is transfected into the HeLa cell line [137]. Also, RNA-seq quantification of BLV antisense transcripts has revealed broadly similar expression levels in BLV latently-infected B cell lines compared to the levels observed in their reactivated counterparts following overexpression of Tax, suggesting that, unlike for the 5’LTR, Tax does not transactivate the BLV 3’LTR antisense promoter activity in vivo.

### Role of BLV antisense transcripts

Precise function of BLV AS1-S, AS1-L and AS2 antisense transcripts is currently unknown. Although the presence of a potential open reading frame has been described for AS1-S and AS1-L [137], experimental evidence for the expression of a protein is still lacking. Alternatively, high-throughput sequencing of nuclear *versus* cytoplasmic RNA fractions has revealed that antisense transcripts are mainly localized in the nucleus, suggesting a potential role of long non-coding RNA (lncRNA). In the HTLV-1 retrovirus, it has been shown that the antisense transcript *HBZ*, also mainly localized in the nucleus [138], supports proliferation of infected T cells by upregulating host genes implicated in cell cycle progression and survival, such as the *surviving* gene [139, 140]. More recently, *HBZ* transcript has been shown to inhibit 5ʹLTR promoter activity by directly interacting with the LTR, resulting in the displacement of the basal RNAPII transcriptional machinery [141]. Due to the close biological and structural similarities between both the BLV and HTLV-1 retroviruses, this suggests that BLV antisense transcripts may support similar functions as HBZ transcripts. Moreover, antisense transcription itself, rather than antisense transcripts or the potentially translated proteins, has been suggested to take part in BLV-induced tumorigenesis [142]. Indeed, stranded RNA-seq analysis of bovine and ovine B-cell tumors has identified the presence of fusion transcripts between AS1-L or AS2 and the host cell genome, resulting in the alteration of the transcribed host genes [142]. Similar observations have been reported in the context of HTLV-1 [142, 143].

### Regulation of BLV antisense transcripts expression

Compared to the BLV sense transcripts, viral antisense transcripts are constitutively expressed, albeit at low level, in ovine and bovine PBMCs either at the asymptomatic or leukemic stages of infection and their expression is correlated with the viral load [137, 142]. Moreover, ChIP-qPCR experiments in the BLV latently-infected cell lines L267 and YR2 and in ovine PBMCs have revealed that the 3’LTR promoter is associated with the presence of activating histone marks, in agreement with the constitutive 3ʹLTR promoter activity [70, 98]. However, the proportion of each transcript is not similar, with AS1-S being the most expressed, and AS2 the less expressed [137]. Interestingly, by a modified 5’RACE assay, AS1-L has been shown to be cleaved by several BLV miRNAs (mainly miR-B1-3p, miR-B4-3p, miR-B2-5p and miR-B2-3p) in the cytoplasm [137]. However, the importance of this cleavage in the regulation of the proportion of AS1 transcripts is uncertain, as the majority of the transcripts does not overlap the miRNA cluster (and is thus not subjected to cleavage) and is nuclear [137]. Instead, a mechanism of transcriptional interference between the RNAPII and RNAPIII complexes transcribing convergently from the 3ʹLTR and the miRNA cluster respectively, has been suggested to regulate the proportion of antisense transcripts [98, 137].

## Conclusions

**Fig. 2:**
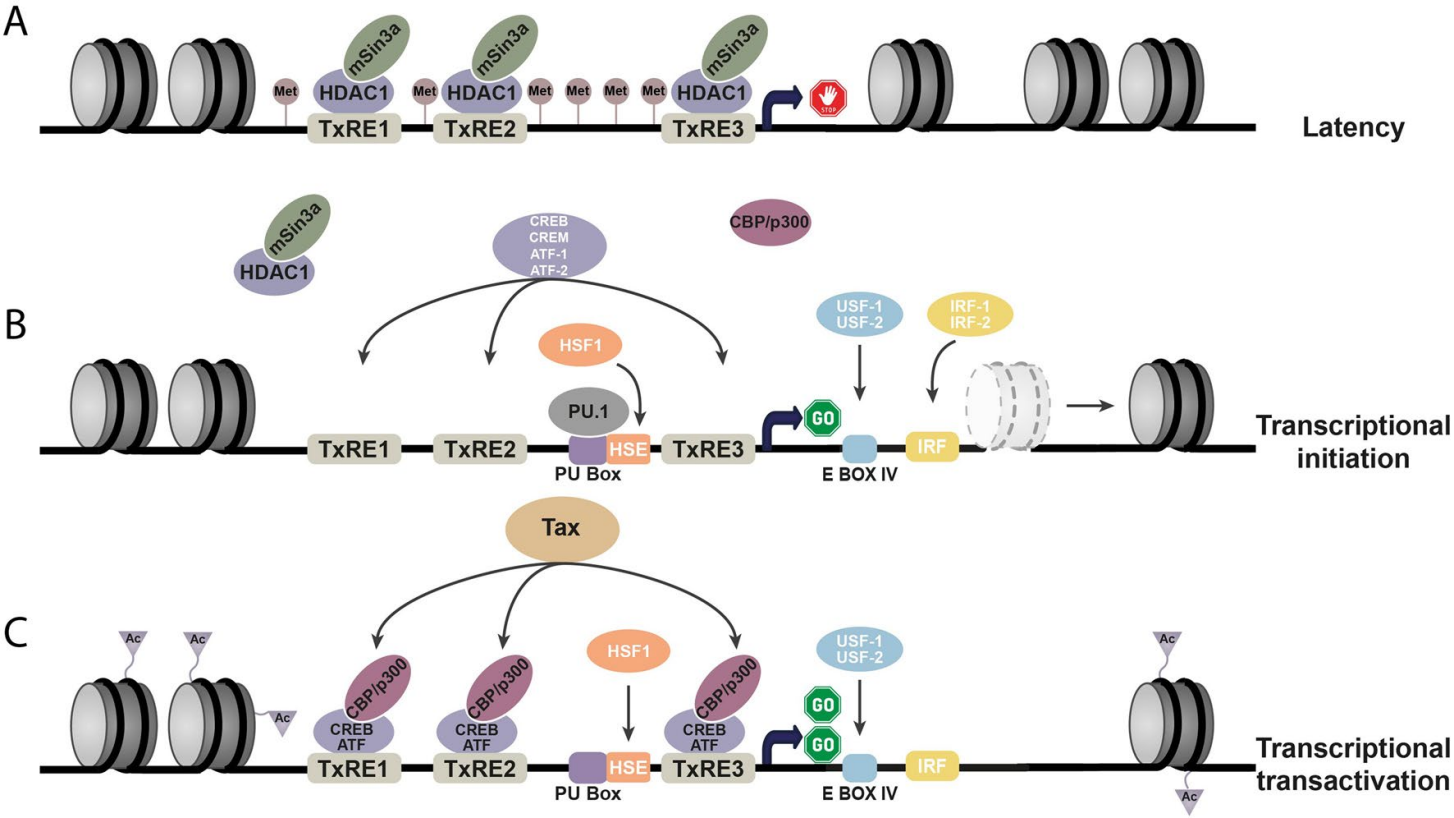
Model of BLV 5ʹLTR latency and transcriptional activation.** A** During latency, the BLV 5ʹLTR promoter activity is repressed by genetic (not shown) and epigenetic mechanisms. These epigenetic mechanisms include the hypermethylation of the DNA and the hypoacetylation of the histone tails due to the recruitment of the histone deacetylase 1 (HDAC1) and the co-repressor mSin3A. **B** Transcriptional initiation might occur by external stimuli or the use of epigenetic drugs. The pioneer transcription factor PU.1 could participate in the opening of the chromatin, increasing the accessibility of *cis*-regulatory elements to other transcription factors such as IRFs, USFs and CREB/ATF factors and the associated co-activators CBP/p300. **C** Once the transcription is initiated, the viral transactivator Tax is produced and directly interacts with CREB/ATF factors increasing their binding affinity for the Tax-responsive elements (TxREs), thereby resulting in strong expression of the viral genes

In the present review, we highlighted the different factors regulating BLV genome expression, from *cis*-regulatory elements present in each BLV promoter to epigenetic modifications regulating the chromatin status of the provirus. Taking all these factors and regulations into account, we propose a model for the reactivation of the BLV 5’LTR promoter from the latency state (Fig. 2). During latency, the fraction of infected B cells containing replication-competent BLV proviruses is maintained transcriptionally silent due to a globally repressive epigenetic environment, which includes: (i) the presence of a nucleosome near the transcription start site, (ii) DNA methylation at different CpG positions, and (iii) hypoacetylation of the histone tails due to the presence of HDAC1 and mSin3a on the E-boxes located in the TxREs (Fig. 2A). Separately or in combination, these epigenetic features characterize a closed chromatin conformation which favors the repression of BLV gene expression. During chronic infection, BLV-infected cells are mainly latent, however, BLV spontaneous reactivation has been previously reported in cattle chronically infected by BLV [61]. The external stimuli involved in BLV reactivation still need to be identified but might be induced by stress, as suggested by Jaworski and colleagues [61]. In the case of HTLV-1, changes in glucose and oxygen concentrations were reported to induce viral reactivation [144] and might thus constitute factors involved in BLV reactivation. These external stimuli, as well as epigenetic drugs, could lead to the activation of the B-cell receptor or other cellular pathways resulting in the sequential action of a cascade of transcription factors involved in BLV reactivation. Through the ability of PU.1 to bind DNA motifs despite a relatively close chromatin environment, the macrophage-specific and B cell-specific pioneer transcription factor PU.1 [54, 145] could participate to the eviction of the repressive nucleosome [146] located downstream of the transcription start site [147], thereby opening the chromatin (Fig. 2B). Once the chromatin opened, IRFs, USFs, HSF1 and CREB/ATF factors could be recruited to the BLV promoter, thereby initiating transcription. The acetyltransferases CBP and p300 could be recruited by either PU.1 [148, 149], or the weakly bound CREB/ATF factors, reinforcing the open chromatin state by acetylating the H3 and H4 histone tails. As transcription is initiated, the first molecules of Tax are produced inducing transactivation of the BLV promoter mainly through interaction of Tax with the CREB/ATF factors increasing their binding affinity to the TxREs and subsequently strong expression of BLV genes (Fig. 2C).

As described in this review, numerous factors are involved in BLV latency and understanding the precise molecular mechanisms and pathways impacting viral expression is important to develop appropriate antiviral therapies. Indeed, developing latency reversing agents would stimulate viral expression and the immune clearance of infected cells. Several DNA demethylating agents and HDACis have been shown to induce BLV transcription in latently-infected cell lines [38, 92, 93] and thus could constitute promising candidates for potential future therapeutic strategies against BLV. In particular, one of the HDACis used in these studies, valproate, has been tested in vivo and has been shown to efficiently reduce the viral load and BLV-induced leukemia in sheep [91]. However, the infection could not be eradicated and development of chemoresistance has been observed [91, 150]. As the need of a treatment is thus still present, several epigenetic drugs reported to be used in cancer therapies [151] could be tested for BLV infection. Importantly, one chemical compound could not be sufficient to eradicate BLV, suggesting that combinatory approaches could improve the therapeutic potential, as previously reported by our laboratory for HIV-1 [152]. Since the HDACi valproate has exhibited great promises for BLV reactivation, it should be tested in combination with DNA methylation inhibitors or other non-epigenetic compounds targeting the mechanisms involved in BLV gene regulation. Alternatively to latency reversing agents, a unique report has identified violaceoid E as a compound repressing BLV expression and host proto-oncogenes by interfering with the transactivating function of Tax [153]. Although the antiviral effects were not strong and were only studied in ex vivo cell cultures, the therapeutic potential of violaceoid E should be further investigated. However, these latency reversing agents might be too expensive for an application at large-scale and could thus be more specifically use in countries with low BLV prevalence or for the selection of valuable cattle breeds.

Compared to other well studied retroviruses such as HIV-1 [154] and HTLV-1 [155], a limited number of *cis*-acting elements have been identified in the BLV promoter region, suggesting the existence of additional *cis*-regulatory elements that need to be identified in the BLV genome. In addition, as reported by Pluta et al. mutations in the BLV LTR occur naturally in BLV-infected cattle and lead to the formation of putative binding sites such as the MYC-associated zinc finger protein (MAZ) site [156]. Therefore, further studies are required to identify potential important *cis*-regulatory elements involved in BLV transcriptional regulation. Moreover, the role of most factors identified so far has been determined using cellular models, such as reporter constructs that, although remaining highly valuable for mechanistic studies, do not recapitulate the complexity of the natural host. Therefore, future research focusing on the function of each factor should be performed in more physiologically relevant models of BLV infection such as BLV-infected cell lines and ultimately in the context of the sheep experimental model or the natural host. The ability of studying BLV infection in the context of its natural host should be further exploited to increase our understanding of BLV gene expression regulation and pathogenesis in vivo and hopefully to translate the promising findings to the closely related retrovirus HTLV-1.

## Appendix

(See Table 1)

**Table 1:** Characteristics of the genomic elements presented in Fig. 1﻿. Genomic coordinates are given relative to the YR2 proviral genome (GenBank KT122858.1). Genomic coordinates in italic are given for the 3’LTR. Nucleotides in bold indicate differences between mature miRNA sequences described in [118] and those in the online database miRBase (https://www.mirbase.org/)

	+ 1 = 1st nt of sense TSS		+ 1 = 1st nt of 5'LTR		Sequence (Positively oriented)	Size (bp)	Reference
Name	Start	End	Start	End			
Proviral genome	− 211	8509	1	8720		8720	KT122858.1
LTR	− 211	320	1	531		531	[63]
U3	− 211	− 1	1	211		211	[63]
*U3*	*7978*	*8188*	*8190*	*8400*		*211*	[63]
TxRE1	− 164	− 143	48	68	CAGACAGAGACGTCAGCTGCC	21	[63]
CRE1	− 157	− 149	55	62	AGACGTCA	8	[40]
E-Box1	− 151	− 145	61	66	CAGCTG	6	[40]
TxRE2	− 139	− 118	73	93	AAGCTGGTGACGGCAGCTGGT	21	[40]
CRE2	− 132	− 124	80	87	AGACGTCA	8	[40]
E-Box2	− 126	− 120	86	91	CAGCTG	6	[40]
PU.1/Spi-B	− 95	− 83	117	128	AAAGGGGAAGTT	12	[49]
HSE	− 86	− 76	126	135	TTTCCCGAAA	10	[58]
CAAT box	− 97	− 91	115	120	CCAACT	6	[63]
GRE	− 74	− 59	138	152	TCCACACCCCGAGCT	15	[25]
TxRE3	− 64	− 43	148	168	GAGCTGCTGACCTCACCTGCT	21	[40]
CRE3	− 57	− 49	155	162	AGACGTCA	8	[40]
E-Box3	− 51	− 45	161	166	CACCTG	6	[40]
TATA box	− 43	− 38	169	173	GATAA	5	[63]
R	1	234	212	445		235	[63]
*R*	*8189*	*8423*	*8401*	*8633*		235	[63]
TSS sense	1		212				[63]
DAS	146	210	358	421		64	[63]
E-Box4	173	178	384	*389*	CACGTG	6	[63]
*E-Box4*	*8362*	*8367*	*8573*	*8578*	*CACGTG*	*6*	[63]
*MTE*	*8405*	*8416*	*427*	*438*	*CAAGCCAGACGC*	*12*	[98]
*DPE*	*8406*	*8410*	*428*	*432*	*AGACG*	*5*	[98]
*TSS2 antisense*	*8380*		402				[136]
U5	235	320	446	*531*		85	[63]
*U5*	*8424*	*8509*	*8634*	*8720*		*85*	[63]
*TSS1 antisense*	*8439*		*8650*				[136]
IRF	250	265	461	476	TACTTTCTGTTTCTCG	16	[63]
*IRF*	*8439*	*8454*	*8650*	*8665*	*TACTTTCTGTTTCTCG*	*16*	[63]
*BRE*	*8475*	*8481*	*497*	*503*	*GGGCGCC*	*7*	[98]
CTCF	285	303	496	514	TGGCCGCTAGAGGGCGCCG	14	[70]
*CTCF*	*8474*	*8492*	*8685*	*8703*	*TGGCCGCTAGAGGGCGCCG*	*14*	[70]
miRNA cluster	6186	6700	6397	6910		514	
miR-B1-5p	6186	6207	6397	6417	AGGCUGUGGUGGUGCACUGGCU**U**	22	[118]
miR-B1-3p	6219	6241	6430	6452	UCAGUGUACCAUCACAAGCCUCU	23	[118]
miR-B2-5p	6297	6316	6508	6527	AUGACUGAGUGUAGCGCAGAGA	20	[118]
miR-B2-3p	6331	6352	6542	6563	UGCGUGUCGCUCAGUCAUUUU**U**	22	[118]
miR-B3-5p	6425	6446	6636	6657	AUCCCCCUGCCAGCGUUGGUC**U**	22	[118]
miR-B3-3p	6459	6481	6670	6692	UAACGCUGACGGGGGCGAUUUCU	23	[118]
miR-B4-5p	6494	6515	6705	6726	GCGGGAGGCUCUGGUGCUGG	22	[118]
miR-B4-3p	6533	6556	6744	6767	UAGCACCACAGUCUCUGCGCCUUU	24	[118]
miR-B5-5p	6647	6669	6857	6880	AGGAAGGUUGUGGCUCAGAGGU	23	[118]
miR-B5-3p	6678	6700	6889	6911	CUCGAGCCGCAACCUCCCUUUCU	23	[118]

## Data Availability

Not applicable.

## Abbreviations

5-azaC: 5-Azacytidine
5-azadC: 5-Aza-2ʹ-deoxycytidine
5’RACE: 5ʹ rapid amplification of cDNA Ends
ABF-1: Activated B-cell factor 1
Ago: Argonaute
AP-1: Activator protein-1
AP-4: Enhancer-binding protein 4
ATF: Activating transcription factor
BLV: Bovine leukemia virus
BRE: TFIIB-recognition element
bZIP: Basic-region leucine zipper
C/EBP: CCAAT/enhancer binding protein
CaMK: Calcium/calmodulin-dependent kinase
cAMP: Cyclic adenosine monophosphate
CBP: CREB binding protein
ChIP: Chromatin immunoprecipitation
CRE: CAMP-response element
CREB: CRE-binding protein
CREM: CRE modulator
CTCF: CCCTC binding factor
DAS: Downstream activator sequence
DNMT: DNA methyltransferase
DPE: Downstream promoter element
E: Glutamic acid
E-box: Enhancer box
EBL: Enzootic bovine leukosis
EMSA: Electrophoretic mobility shift assays
GNAT: General control non-repressed 5 protein-related N-acetyltransferases
GRE: Glucocorticoid response element
HAT: Histone acetyltransferase
HBP1: High mobility group box transcription factor 1
HDAC: Histone deacetylase
HDACi: HDAC inhibitor
HDMT: Histone demethylase
HMT: Histone methyltransferase
HSE: Heat shock element
HSF1: Heat shock factor 1
IRF: Interferon regulatory factork lysine
lncRNA: Long non-coding RNA
LTR: Long terminal repeat
K: Lysine
Mad1: Max dimerization protein 1
Mad4: Max dimerization protein 4
MAPK: Mitogen-activated protein kinase
Max: MYC associated factor X
MAZ: MYC-associated zinc finger protein
miRNA: MicroRNA
mSin3A: Mammalian SIN3 transcription regulator family member A
MTE: Motif Ten Element
MYST: Named for its founding members MOZ, YBF2/SAS3, SAS2, and Tip60
NaB: Sodium butyrate
NFAT: Nuclear factor of activated T-cells
P-TEFb: Positive-transcription elongation factor b
PAS: Polyadenylation signal sequence
PBMC: Peripheral blood mononuclear cells
PKA: Protein kinase A
PKC: Protein kinase C
PMA: Phorbol-12-myristate 13-acetate
pre-miRNA: Precursor-miRNA
RISC: RNA-induced silencing complex
RNAP: RNA polymerase
TBP: TATA-box binding protein
TPX: Trapoxin
TSA: Trichostatin A
TxRE: Tax-responsive element
USF-1: Upstream stimulatory factor 1
USF: Upstream stimulatory factor 2
WT: Wild-type
